# Data-driven insights into interhospital care fragmentation: Implications for health policy and equity among older adults

**DOI:** 10.1371/journal.pone.0316829

**Published:** 2025-02-04

**Authors:** Somayeh Ghazalbash, Manaf Zargoush, Vedat Verter, Dan Perri

**Affiliations:** 1 Management Analytics, Smith School of Business, Queen’s University, Kingston, Ontario, Canada; 2 Health Policy and Management, DeGroote School of Business, McMaster University, Hamilton, Ontario, Canada; 3 Division of Gastroenterology and Critical Care Medicine, Department of Medicine, McMaster University, Hamilton, Ontario, Canada; 4 Divisions of Clinical Pharmacology & Toxicology and Critical Care Medicine, Department of Medicine, McMaster University, Hamiton, Ontario, Canada; Cardiff University, UNITED KINGDOM OF GREAT BRITAIN AND NORTHERN IRELAND

## Abstract

**Objective:**

To determine factors leading to interhospital care fragmentation (ICF); evaluate how ICF affects rehospitalization costs, length of stays (LOS), and delayed discharge; and analyze ICF disparity among equity-seeking groups.

**Materials and methods:**

We used a 13-year retrospective cohort of older adults (65+) in Ontario, Canada. Utilizing multivariable logistic regression, we identified characteristics associated with ICF and determined its association with outcomes.

**Results:**

Discharge to facilities except home and homecare and travel distance were the strongest risk factors for ICF. Patients were less likely to experience ICF if they were older, frail, or had multiple comorbidities. ICF was strongly associated with an increase in the daily costs of readmission. Moreover, the risks of a prolonged LOS after ICF and delayed discharge were higher among returning surgical patients. The rural residency was a source of health inequality.

**Conclusions:**

ICF exacerbates health disparities and worsens patient outcomes. Our study identified several risk factors associated with ICF, some of which are controllable, paving the way for interventions to mitigate this issue. To promote health equity and reduce adverse outcomes, policymakers should focus on policies for reducing care discontinuity, particularly addressing the controllable risk factors.

## 1. Introduction

Care fragmentation (CF) occurs when patients receive care from various providers across multiple care settings without efficient coordination and integrated clinical information [1, 2]. Interhospital care fragmentation (ICF) occurs when a patient is readmitted to a different hospital (non-index hospital) from the one they were originally discharged from (index hospital) [2]. An index hospital refers to the hospital where a patient first received care, while a non-index hospital is any hospital other than the index hospital. This form of fragmented care is a prevalent example of CF, making up approximately 20–30% of unplanned readmissions [3, 4]. Because hospitals are less likely to share key patient information (e.g., medical history, complications, progress notes, diagnostic test results, and in-hospital therapies/interventions), ICF leads to negative impacts on formal and informal informational continuity [3, 5]. Furthermore, when a patient is readmitted to a hospital different than the index hospital, it poses substantial challenges to post-discharge coordination between the index and non-index hospitals, thereby negatively affecting patient outcomes like post-discharge mortality, service utilization, and rehospitalization [6, 7].

From a broader perspective, the World Health Organization (WHO) envisions integrated care as a seamless continuum of health services catering to individuals’ needs throughout their lifespan [8]. Under its ICOPE (Integrated Care for Older People) approach, the WHO advocates for health and social services that integrate governance and service models to prevent avoidable declines in older people’s capacity and functional ability. Central to this philosophy are comprehensive care plans, unified data-sharing systems, and supportive governance, among other facets [9]. However, ICF contradicts the WHO’s ICOPE vision, particularly negatively affecting older adults by moving away from the principle of integrated, person-centered care.

Exploring the roots of ICF reveals a myriad of factors, some of which are patient-driven, while others originate from care providers or hospital systems [10]. For instance, patients might opt for surgical procedures at one hospital but seek medical care at another due to specialized services or convenience. This multitude of patient choices, coupled with the lack of seamless transfer of medical documentation between facilities, worsens the fragmentation. A particularly vulnerable group consists of older patients who are unable to effectively communicate their medical histories due to cognitive or communication impairments. Given these dynamics, it is imperative to investigate ICF’s underlying factors and ramifications. Such analysis will provide healthcare professionals with insights to devise strategies to enhance care coordination, bolster patient outcomes, optimize resource utilization, and offer personalized care.

The discussion of ICF also highlights a troubling aspect of healthcare disparities among different social groups, which has been recognized as a major challenge to public health [11, 12]. Evidence suggests a higher prevalence of ICF among certain sociodemographic groups, such as racial and ethnic minorities [13] and individuals in rural locales [3], attributing to their increased barriers in accessing and coordinating care. This amplifies the necessity to investigate the issue of disparities in ICF among equity-seeking groups to unearth and address health inequalities, thereby fostering equitable healthcare access. Previous studies have shown the association of ICF with patient outcomes in ambulatory care settings [14, 15]; however, risk factors for ICF and its association with patient outcomes in acute care settings have not been thoroughly investigated [2]. The existing studies have focused on specific patient populations (e.g., cardiac or surgical patients) [2, 16, 17]. These studies have not been differentiated by the reason for readmission, leading to inconsistent conclusions about the relationship between ICF and patient outcomes [2].

Moreover, little is known about the extent and nature of health disparities among patients with ICF. Only a few studies have explored the role of socioeconomic factors such as household income and insurance status, primarily focusing on common relative measures such as the odds ratio [16, 18]. Although relative measures may be informative regarding the strength of the association between risk factors and outcomes, absolute measures such as prevalence or risk difference reflect the actual public health burden. By considering both types of measures, we obtain a more comprehensive view of the magnitude and practical significance of health inequalities. To our knowledge, no studies have comprehensively quantified the impact of socioeconomic risk factors on ICF. To close the gaps, this study aimed to determine potential risk factors for ICF and its association with adverse outcomes like prolonged hospital stay, delayed discharge, and increased hospitalization costs. The study also aimed to investigate the racial and socioeconomic disparities regarding ICF among various equity-seeking groups using several absolute and relative disparities measures.

## 2. Materials and methods

### 2.1. Data and study population

A retrospective cohort analysis was conducted on individuals aged 65 and above in Ontario, the largest province in Canada, from April 2004 to March 2017, and it was accessed for analysis between April 1^st^, 2023, and April 1^st^, 2024. The study data was derived from two databases at the Institute for Clinical Evaluative Sciences (IC/ES). The Discharge Abstract Database (DAD) offers detailed information about patients’ acute care services, including diagnoses and length of hospitalization for each visit. The patients’ sociodemographic characteristics were extracted using the Registered Persons Database. Patient data were encrypted, ensuring that authors could not identify individual participants during or after data collection. The initial dataset contained 6,039,054 observations collected from 1,741,830 patients. We excluded patients with the following characteristics: (i) patients who died during the index hospital visits; (ii) patients who were transferred to another acute care hospital; (iii) patients who left the hospital against medical advice during any of the admissions; and (iv) patients who had a planned readmission. A total of 3,748,775 observations were found eligible to be included in this study. We further restricted our cohort to include only patients with at least one readmission within 30 days of discharge, yielding 963,320 unique observations (S1 Fig). The study was reviewed and approved by the Hamilton Integrated Research Ethics Board (HiREB). The initial ethics approval for this study was granted on October 17, 2018, under Project ID 5472. The study has undergone annual ethics renewals as required, with approvals granted for each subsequent year. The most recent ethics approval was issued on September 17, 2024, ensuring compliance with ethical guidelines throughout the study period.

### 2.2. Outcome variables

Our primary outcomes were (i) delayed discharge (DD), (ii) prolonged length of stay (LOS), and (iii) hospitalization costs of readmissions within 30 days. Delayed discharge, also known as Alternate level of care (ALC) in Canada, occurs when patients continue to occupy hospital beds despite no longer requiring the level of care provided, often while awaiting placement in community-based services [19]. The occurrence of delayed discharge is assessed as a binary outcome, as whether or not a patient is designated as ALC. The designation of a patient as ALC typically involves a multidisciplinary team in a healthcare setting, primarily led by physicians, in consultation with nurses, social workers, and sometimes allied health professionals. This designation is based on a comprehensive assessment of the patient’s medical condition, care needs, and the most appropriate setting to meet those needs [20]. Hospitalization cost was defined as the average daily expenses associated with the patient’s future return to the hospital within 30 days, measured using the patient’s utilization of hospital resources at the end of each visit divided by the readmission LOS. Then, we dichotomized the daily patients’ costs as “excessive costs” and “in-excessive costs,” depending on whether a patient incurred a cost higher than the mean value. The LOS was categorized as “prolonged LOS” if it exceeds the average LOS and “non-prolonged LOS” otherwise.

### 2.3. Covariates

The primary variable was the type/location of unplanned readmission within 30 days of index admission, dichotomized as the index and non-index hospital. We differentiated between patients who were readmitted to a hospital either for the same or a different reason if the index hospital had the necessary specialists to treat the condition. We defined the reason using the patient’s primary diagnosis, coded by ICD-10 (International Statistical Classification of Diseases and Related Health Problems, Tenth Revision) [21]. Patients were classified as having the same reason for hospitalization if their first three characters of the ICD-10 code were the same on their rehospitalization. We focused on the first three characters of the ICD-10 because they describe the general type of the patient’s injury or disease [22].

The covariates were classified into four categories: (i) patient demographics (age, sex), (ii) socioeconomic status (marginalization measured using the Ontario marginalization index [23], rural/urban residency, the distance between patients’ residency and hospital), (iii) clinical variables (multimorbidity score measured by Charlson–Deyo Comorbidity Index (CDCI) [24], frailty score measured by Hospital Frailty Risk Score (HFRS) [25], the most common interventions, and surgical services during index admission), and (iv) administrative variables (hospital LOS, discharge disposition categorized as home, home with support, and other facilities, and admission to a special care unit (SCU) such as cardiac care or intensive care unit).

### 2.4. Statistical analysis

We summarized the distribution of patient characteristics in terms of the readmission type (fragmented versus non-fragmented) using Fisher’s exact and chi-square tests. Descriptive statistics were reported using measures of frequency for categorical variables. A multivariate logistic regression was conducted to identify the most important risk factors for ICF. Subsequently, we considered ICF an independent variable and performed separate multivariate logistic regressions to assess the association between ICF and three outcomes (i.e., delayed discharge, LOS, and rehospitalization costs) while adjusting for the abovementioned covariates.

We also investigated if there was a significant interaction between receiving surgical services at admission and patient outcomes. This is due to the previously reported association regarding the increased risk of adverse outcomes for surgical patients who are rehospitalized at a different care setting [26]. The absence of multicollinearity was examined through the predictors’ VIF (variance inflation factor). Missing values for continuous variables were imputed by their mean [27]. In our study, the only continuous variable with missing values was distance, which accounted for 2.2% of the data (21,536 observations). All tests were two-sided with a statistical significance level of α = 0.05, and all estimates were presented with their 95% confidence intervals (CI). All statistical analyses were performed using the R version 4.2.1 package *tidyverse* [28].

### 2.5. Robustness analysis

To assess the robustness of our results, we conducted several separate robustness analyses. First, we substituted hospitals with the corresponding facility (i.e., the hospital network); hence, we did not distinguish between the hospitals that belonged to the same healthcare system and might have a connected electronic health record. In Canada, healthcare is primarily delivered through a publicly funded system, where provincial and territorial governments oversee the administration of hospitals and healthcare facilities. Many hospitals within a province may be part of larger healthcare networks or regional health authorities, allowing coordinated care across multiple facilities. Hospitals often share connected electronic health record (EHR) systems in such networks, enabling seamless data exchange and integrated patient care across institutions. In this context, our analysis treated hospitals within the same network as a single facility, reflecting how these institutions collaborate and share patient information.

Second, we performed additional analyses using regression standardization and Doubly robust analysis to ensure the robustness of our results in identifying the risk factors associated with ICF while adequately controlling for confounding effects. Unlike standard logistic regression, which estimates conditional effects, regression standardization provides marginal effects by averaging the risk over the distribution of confounders in the population. This approach leads to more interpretable results across the entire population and helps account for interactions between risk factors and confounders, ensuring that the estimates reflect the overall effect, even in complex models [29]. Additionally, we employed Doubly robust methods to address potential sources of bias by combining propensity score modeling and regression adjustment [30, 31]. Specifically, we utilized Inverse Probability of Treatment Weighting (IPTW). This involved initially calculating the propensity of exposure to the risk factor of interest and then creating weights as the inverse of the propensity score to ensure an equal distribution of confounding variables across exposed and unexposed groups in a pseudo-population. In cases where subjects have a very low probability of exposure, we utilized weight stabilization or weight truncation (trimming) to address the instability caused by extreme weights [32]. By integrating this approach with regression adjustment in doubly robust analysis, we can obtain valid estimates of the association between the risk factors and ICF, even if either the exposure model or the outcome model is misspecified.

Third, we repeated the analyses using a propensity score matching (PSM) method to assess our results’ robustness after reducing confounding effects when estimating the effect of ICF on patient outcomes. To match pairs, we used a greedy nearest-neighbor matching approach. This involved randomly selecting a patient who had been readmitted to a different hospital and then matching them to a patient who had been readmitted to the index hospital with the closest propensity score [33]. We further employed the Doubly Robust Assessment method [30]. For standardization, we used the *stdReg* package [29], and for propensity score analysis, we employed the *MatchIt* [34] and *WeightIt* [35] packages in R. Fourth, we varied the cut-off point used to define prolonged LOS and daily costs to the median and 90^th^ percentile, as suggested by other studies [21, 36], to test the sensitivity of our findings to changes in the cut-off point. Finally, we employed two alternative methods for addressing missing data: eliminating the missing values and replacing them with the median instead of the mean.

### 2.6. Measuring health inequalities

There is much debate about how to measure inequalities in the population. Therefore, we considered both absolute and relative measures of risk to interpret health inequalities and to provide comprehensive information about their nature and impact [37]. We focused on “prevalence” and “risk difference” as the absolute measures and “frequency ratio,” “attributable risk proportion” (ARP), and “population-attributable risk proportion” (PARP) as the relative measures of inequalities [38, 39]. These are the most commonly used measures for equality comparisons [38, 39]. The absolute measures of risk provide information on the actual number of ICF cases in each equity-seeking group, helping to understand the public health impact of inequality. On the other hand, relative risk measures provide information on the magnitude of the difference in the ICF risk across groups, helping to understand the strength of the association [38, 39]. We quantified all measures for the subgroups defined by three socioeconomic characteristics: sex (female vs. male), residency (rural vs. urban), and ethnic minority (visible minority vs. majority).

The prevalence of an outcome is the proportion of individuals in a population who have the outcome at a given point in time [40]. The risk difference is the absolute difference in the risk of an outcome between two groups [40]. The prevalence ratio shows how much greater (or smaller) the prevalence of an outcome is in one group to the reference category. The ARP measures the contribution of the exposure to the risk of the outcome in the exposed group. The PARP estimates the proportion of an outcome in a population that can be attributable to a specific risk factor [40]. In other words, the event would not have occurred in the absence of that risk factor. It accounts for both the prevalence of the risk factor and the excess risk of events associated with the risk factor [40]. The measures of disparity are calculated using the *twoxtwo* Package in R [41].

## 3. Results

### 3.1. Descriptive statistics

S1 Table provides a summary of our descriptive analyses. The study included 963,320 observations of patients aged 65–102 readmitted within 30 days after discharge. Of these patients, 36% were readmitted to a non-index hospital. The proportion of ICF increased significantly over the 13-year study period from 36% in 2005 to 46% in 2017. Overall, the patient characteristics, such as sex, comorbidity, frailty, and minority, were roughly similar among those readmitted to a non-index hospital as opposed to the index hospital. However, patients who experienced ICF were younger (74.5 vs. 75.5), primarily stayed in an SCU during their admission (29.6% vs. 14.2%), more likely to live in rural areas (26.5% vs. 17.4%), had surgery during their initial hospital stay (18.9% vs. 13.8%), and were more frequently discharged to other facilities other than their home or with home support (57.5% vs. 18.7%). Additionally, the hospitals where patients received their initial treatment were significantly closer to their home residency than non-index hospitals (18.2 km vs. 42.0 km). Patients with ICF had higher daily readmission costs (Mean = $1992, 95% CI = $1987–$1996 versus Mean = $1648, 95% CI = $1644-$1651) compared to those with same-hospital readmissions; and the mean of LOS slightly differed between the two groups (Mean = 10.77, 95% CI = 10.72–10.82 versus Mean = 11.09, 95% CI = 11.02–11.16). The results also indicated a slight difference in the delayed discharge rate for patients readmitted to a non-index hospital versus an index hospital (12.4% versus 11.1%).

### 3.2. Risk factors for ICF

**Fig 1:**
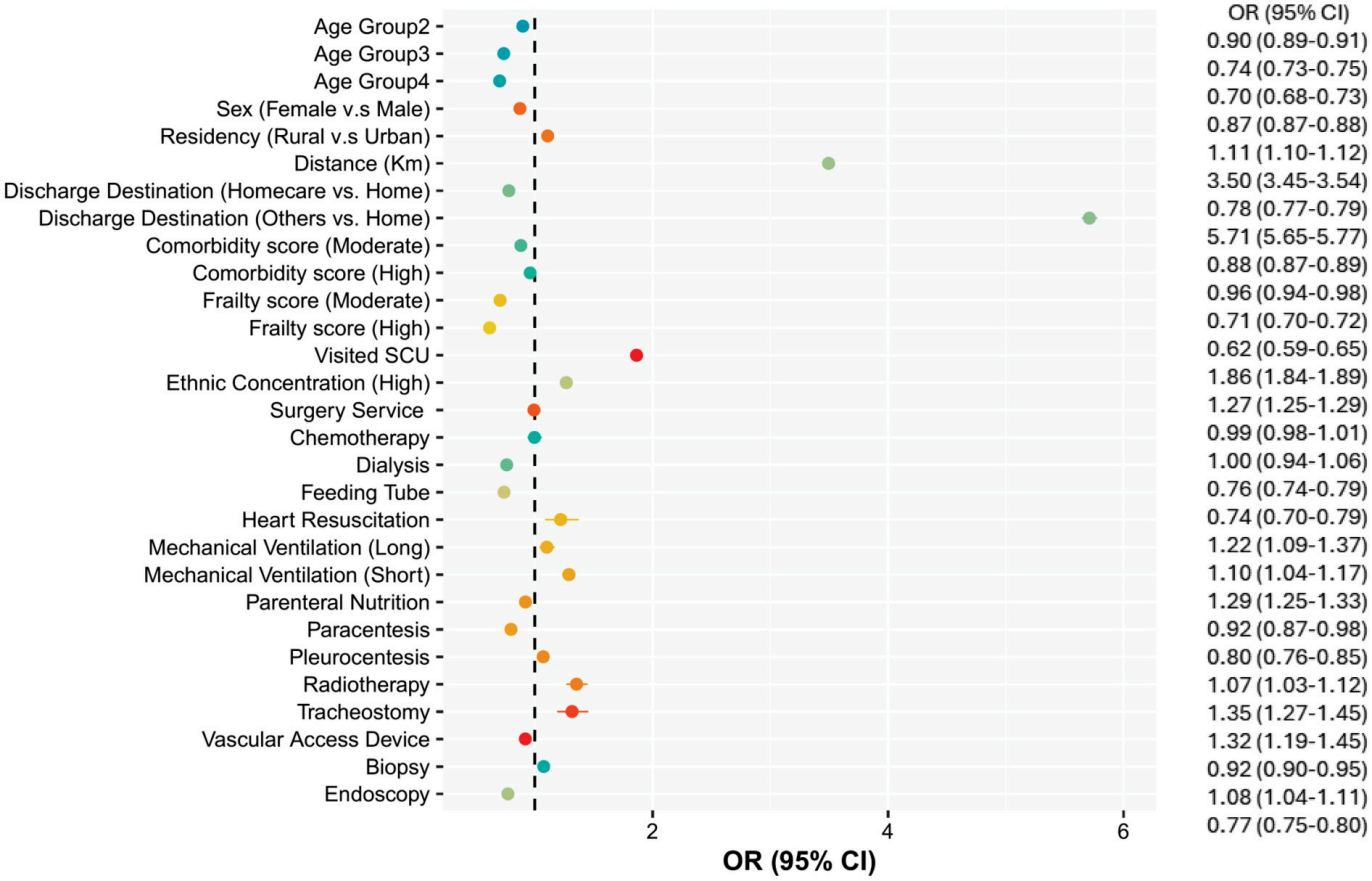
Risk factors associated with ICF. Note: Age Group 1: 65–71 Yrs, Age Group 2: 72–81 Yrs, Age Group 3: 82–91 Yrs, Age Group 4: >91 Yrs.

Fig 1 illustrates the risk factors associated with ICF. The strongest risk factors for ICF were discharge to any facilities except home and homecare (OR = 5.70, 95% CI = 5.64–5.77) and traveling to a long-distance hospital for admission (OR = 3.49, 95% CI = 3.45–3.53). The likelihood of ICF was significantly increased for males (OR = 1.14, 95% CI = 1.13–1.15), rural residents (OR = 1.11, 95% CI = 1.09–1.12), minority groups in terms of ethnicity concentration (highest quartile: OR = 1.26, 95% CI = 1.25–1.28), and those with an SCU stay during index admission (OR = 1.86, 95% CI = 1.84–1.88) compared to their reference groups. In contrast, older patients (very old (>91 yrs): OR = 0.70, 95% CI = 0.67–0.72) with greater complex needs, i.e., high comorbidity burden, i.e., scores greater than 5, (OR = 0.96, 95% CI = 0.94–0.98) and high frailty burden, i.e., scores greater than 15, (OR = 0.61, 95% CI = 0.59–0.64) were more likely to be returned to the index hospital. Those patients who had heart resuscitation, mechanical ventilation, radiotherapy, and tracheostomy interventions in their index admission were more likely to experience ICF.

### 3.3. Impact on patient outcomes

**Fig 2:**
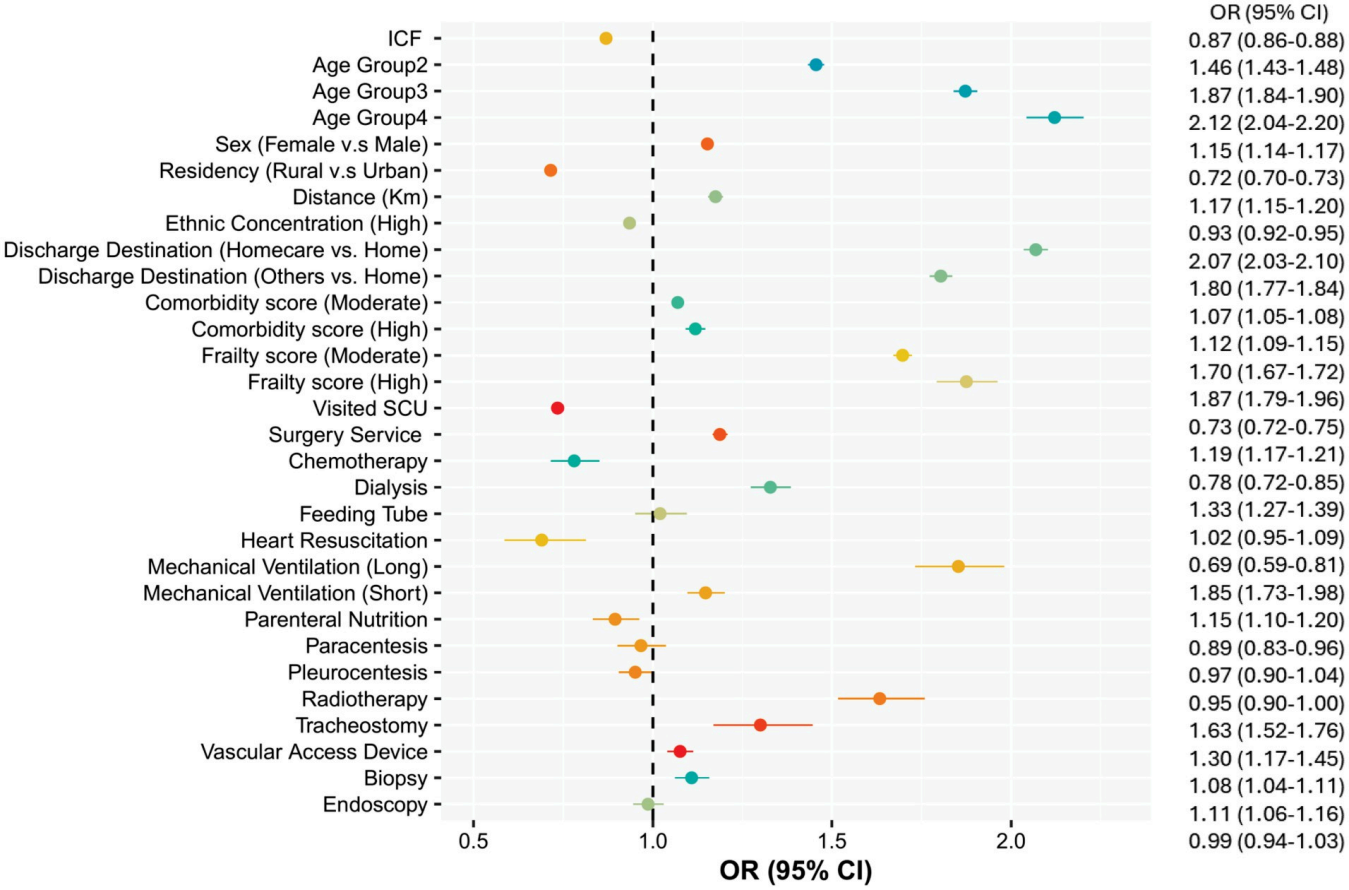
Risk factors associated with delayed discharge (alternate levels of care). Note: Age Group 1: 65–71 Yrs, Age Group 2: 72–81 Yrs, Age Group 3: 82–91 Yrs, Age Group 4: >91 Yrs.

Our results of the association between delayed discharge and ICF (Fig 2) indicate that patients rehospitalized to a non-index were less likely to be designated as DD (OR = 0.87, 95% CI = 0.86–0.88). Interestingly, the interaction between ICF and receiving surgical services at index hospital was significant, such that the risk of DD was significantly higher for those patients who had surgery during their index visit and experienced ICF compared to their reference group (OR = 1.31, 95% CI = 1.28–1.34).

**Fig 3:**
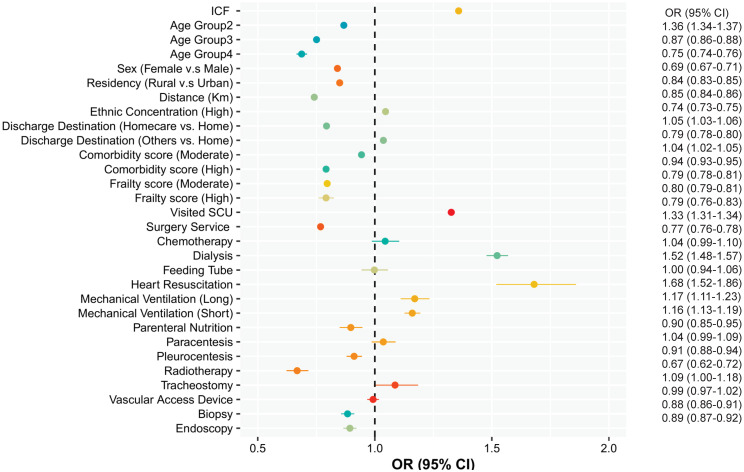
Risk factors associated with the daily cost of readmission. Note: Age Group 1: 65–71 Yrs, Age Group 2: 72–81 Yrs, Age Group 3: 82–91 Yrs, Age Group 4: >91 Yrs.

**Fig 4:**
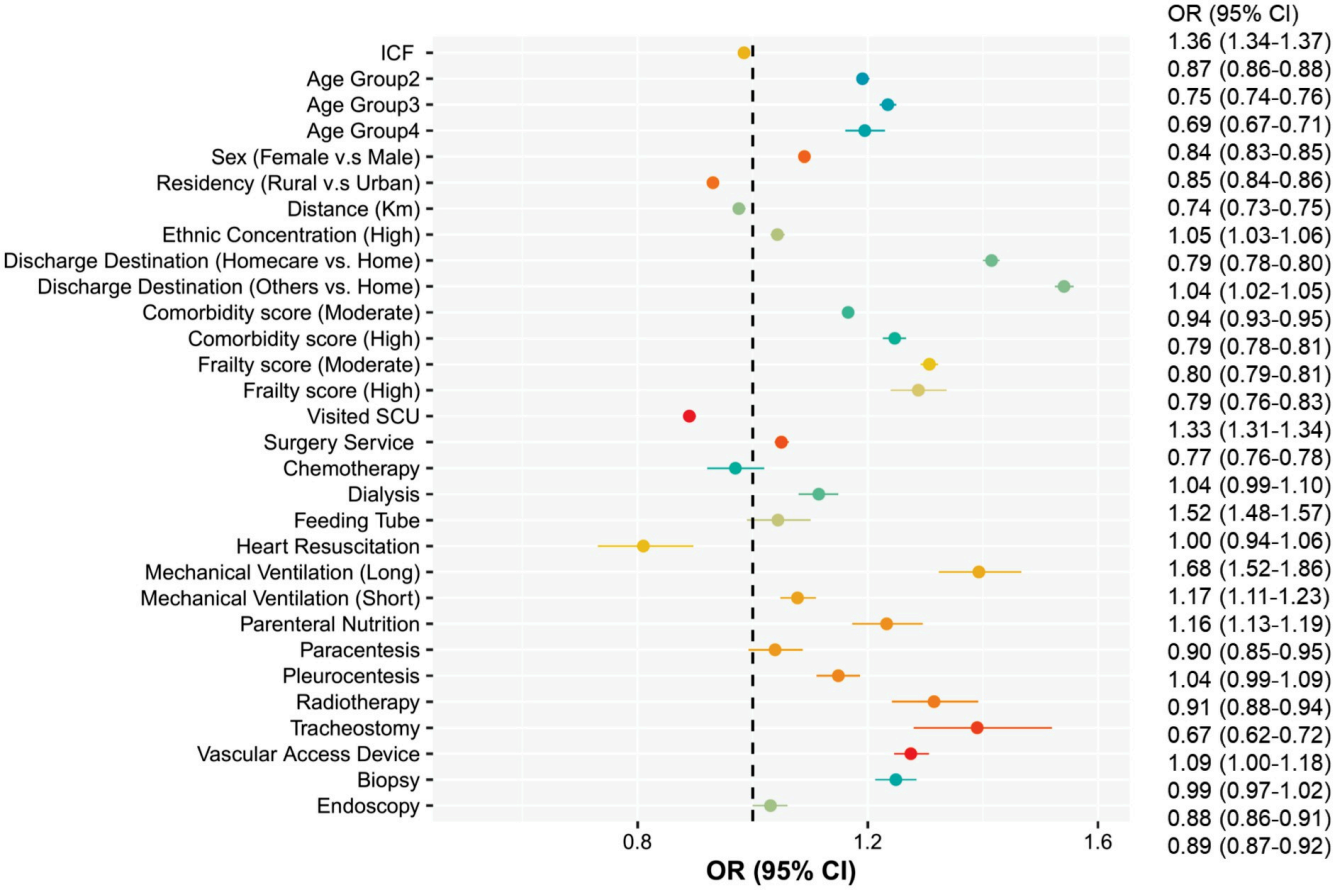
Risk factors associated with prolonged length of stay after readmission. Note: Age Group 1: 65–71 Yrs, Age Group 2: 72–81 Yrs, Age Group 3: 82–91 Yrs, Age Group 4: >91 Yrs.

The multivariate analyses (Figs 3 and 4) also suggest that ICF is strongly associated with an increase in daily readmission cost (OR = 1.36, 95% CI = 1.34–1.38) but not with a prolonged LOS (OR = 0.96, 95% CI = 0.95–0.97). An interaction term between surgical services and ICF was significant for a longer LOS, indicating that the risk of a prolonged LOS after ICF is higher among returning surgical patients (OR = 1.19, 95% CI = 1.17–1.21).

### 3.4. Robustness analysis

To evaluate the robustness of our findings, we investigated whether receiving care from hospitals with the same healthcare system changes the impact of ICF on the outcomes. To this end, we substituted the facility number with the institution number. The robustness analysis resulted in slight changes in the magnitude of the odds ratios, though the direction remained unchanged (S2–S5 Tables).

Upon employing strategies to minimize the impact of confounding on estimating the causal effects of ICF, the odds ratios remained consistent across all three methods: logistic regression, regression standardization, and Doubly robust analysis for almost all risk factors (S6 Table). This demonstrates the reliability of our findings and strengthens the conclusion that the observed associations are stable. However, we observed an anomaly in the receiving surgical services. While regression standardization indicated a significant odds ratio of 1.02 (95% CI: 1.01–1.03), this was not evident in logistic regression and doubly robust analysis. Despite the odds ratio being very close to 1, implying a minimal effect, this discrepancy may be because regression standardization provides population-wide causal interpretations through estimating marginal effects, whereas the other methods focus on conditional estimates.

We further examined the relationship between ICF and patient outcomes using matched data from propensity score matching. After matching, we observed a decrease in the strength of the odds ratios across outcomes (S7 Table). However, the primary interpretation of the findings remained consistent. The only deviation was in the case of length of stay, where the odds ratio became statistically significant after matching (OR: 1.03, 95% CI: 1.00–1.06). Nonetheless, the odds ratio remains very close to 1, indicating a minimal change in effect size. This suggests that while the finding is statistically significant, it may not have a significant clinical impact.

Comparing the results across the different cut-off points used to define prolonged LOS and daily costs suggested slight changes in the magnitude of likelihoods but no change in their directions (${\text{OR}}_{\text{LOS}>90\text{th}\;\text{percentile}}^{30d}=\;0.96$, 95% CI = 0.95–0.97; ${\text{OR}}_{\text{LOS}>\text{median}}^{30d}=0.99$, 95% CI = 0.98–1.00; ${\text{OR}}_{\text{Cost}>90\text{th}\;\text{percentile}}^{30d}=1.37$, 95% CI = 1.35–1.38; ${\text{OR}}_{\text{Cost}>\text{median}}^{30d}=1.43$, 95% CI = 1.42–1.44).

Finally, we employed two alternative methods for addressing missing data: eliminating the missing values and replacing them with the median instead of the mean. This modification did not influence our results, underlining the durability of our findings (S8 and S9 Tables).

### 3.5. Health inequalities

The prevalence of ICF was higher among rural residents than urban residents (rural: 46% vs. urban: 35%), among males compared to females (male: 38% vs. female: 35%), and in the ethnic majority group compared to minority groups (majority: 37% vs. minority: 35%). The absolute risk difference for residency indicates that around 13% more individuals in the disadvantaged population (i.e., rural patients) experience the ICF compared to the advantaged group (i.e., urban patients). The frequency ratio of 1.4 for the residency indicated that the patients living in rural areas have a 40% higher risk of ICF compared to the patients living in urban areas. The ARP and PARP for residency associated with ICF were 27.6% (95% CI: 27.2–28.0) and 7.30% (95% CI: 7.16–7.44). The ARP for residency suggests that around 28% of the cases of ICF in patients living in rural areas can be attributed to rural residency. This finding may be due to multiple factors, including limited access to healthcare, transportation barriers, and higher poverty rates in rural areas [18].

Moreover, the PARP of residency showed that 7.3% of all cases of ICF in the population can be attributed to rural residency. Together, these findings suggest that rural residency is an important contributor to the risk of ICF, both at the individual level (ARP) and the population level (PARP), suggesting that rural residency is a source of health inequality. However, the evaluated inequality measures indicated favorable disparity for female and minority groups, thus not evidencing any marked inequality against these demographics (Table 1). Exploring other dimensions of marginalization, i.e., income and dependency, revealed a contrasting scenario: groups with higher dependency (defined by the ratio of older adults and children to the population aged 15–64 and the percentage not participating in the labor force) [42] and lower income faced unfavorable disparities, unlike the favorable disparity observed for minority groups (ARP_dependency_ = 1.27%, CI = 0.73–1.81; ARP_income_ = 2.21%, CI = 1.53–2.89). These findings highlight the multidimensional nature of marginalization in health inequalities, suggesting a need for a more integrative approach to analyzing and addressing the diverse facets of marginalization beyond just ethnic concentration. Table 1 presents a summary of the measures evaluating health inequalities regarding ICF.

**Table 1:** Inequality measures for three equity-seeking groups (residency, sex, marginalization).

Inequality Measures	*Rural vs*. *Urban*	*Male vs*. *Female*	*Majority vs*. *Minority*
***Absolute Measures***			
Frequency (%)	46.4 vs. 33.6	37.9 vs. 34.5	36.5 vs. 34.9
ARD (%)	12.81	3.32	1.56
***Relative Measures***			
Frequency Ratio	1.38	1.09	1.04
ARP, %, 95% CI	27.6 (27.2–28.0)	8.76 (8.27–9.24)	4.28 (3.57–4.98)
PARP, %, 95% CI	7.30 (7.16–7.44)	4.69 (4.42–4.96)	3.61 (3.01–4.21)
ARP = Attributable Risk Proportion; PARP = Population Attributable Risk Proportion; ARD = Absolute Risk Difference			

## 4. Discussion

### 4.1. Principal findings

We conducted a retrospective study on a large dataset of older Canadians to examine the factors that contribute to ICF, as well as the potential association between ICF and other important outcomes, such as delayed discharge as well as hospitalization costs, and LOS. Our results suggest that more than a third of patients were readmitted within 30 days to a hospital different from an index hospital, with an increasing trend from 2005 to 2017. Of all the factors associated with ICF, discharge to any facility except home/homecare and the distance traveled from the patient’s residency to the hospital were the strongest risk factors. We found evidence of increased rehospitalization costs but no significant effect on LOS when patients were readmitted to a different hospital, supported by robust analyses. In contrast, delayed discharge after readmission was more likely to happen at the index hospitals than at non-index hospitals. Our results indicate that index hospitals are closer to the patient’s home residency on average than the non-index hospitals. Moreover, these patients were older and more complex, with a higher frailty and comorbidity. However, the coexistence of surgical services at an index hospital and readmission to a non-index hospital increased the relative risk of delayed discharge after rehospitalization. We also showed the critical role of socioeconomic factors, such as residency, sex, and ethnic minority, in understanding health disparities that lead to unequal health outcomes, an important area of focus for public health initiatives.

### 4.2. Comparison with previous research

Our results agreed with the literature supporting the importance of continuity of care with the same healthcare institution. In line with previous literature, we found that ICF is associated with higher hospitalization costs following readmission [3, 36, 43]. Existing studies have reported inconsistent patterns between ICF and prolonged LOS. Some studies reported a significant association between prolonged LOS and ICF [3, 26, 43–46]; others found no statistically significant association [47]. Our findings suggest that the rehospitalization LOS is not significantly higher among patients experiencing ICF. We speculate that the limited scope of these studies, focusing on a narrow population, like heart failure patients [3, 43] or surgical patients [26], might cause the observed differences. However, we used a heterogeneous population of older adults over 13 years.

Moreover, we found a significant interaction between surgical services and ICF, suggesting a slightly higher LOS for surgical patients who were rehospitalized at a different hospital, as reported in some studies within this context [26]. Older patients with more complex needs, such as those with a higher burden of frailty and comorbidity, return to index hospitals, which are closer to their home residency than non-index hospitals. Hence, the risk of delayed discharge is higher among those readmitted to an index hospital.

Consistent with previous literature, our findings suggest that younger patients from rural areas have a greater risk of ICF [3]. Further, we found that admission to the special care units, such as the intensive care unit (ICU), coronary ICU, medical ICU, and surgical ICU, during the hospitalization increased the risk of 30-day ICF. Our findings align with previous literature showing a pattern between critical care unit admission and increased risk of 30-day readmission [48]. However, to the best of our knowledge, no studies have investigated the impact of this factor on the 30-day ICF. In addition, we discovered that patients who underwent intubation and mechanical ventilation greater than 96 hours had a higher risk of readmission to a non-index hospital and subsequent longer length of stay, more hospitalization charge, and a higher likelihood of delayed discharge. In agreement with our results, a retrospective study revealed that nearly one in four mechanically ventilated patients in the emergency department were readmitted to the hospital within 30 days [49]. Similar to previous works that revealed the role of socioeconomic factors in ICF [16, 18], this work identified rural residency as the ICF contributor at both individual and population levels and a source of health inequality.

### 4.3. Health policy implications

The findings of this study have important health policy implications, particularly in the context of ICF and its consequences. Identifying distance as the strongest risk factor for ICF highlights the need for policy interventions to improve access to healthcare services, particularly for older patients. Efforts should be made to ensure that individuals living in remote or underserved areas have adequate access to healthcare facilities, reducing the need for patients to travel long distances for care and potentially reducing interhospital transfers. One promising avenue to mitigate the issues of ICF and enhance care coordination is the utilization of Health Information Exchanges (HIEs). HIEs facilitate the sharing of health data from electronic medical records/hospital information systems between hospitals and have been shown to reduce ICF [50], lessen duplication of diagnostic imaging [51], decrease costs [52], and lower the risk of readmissions [53]. Especially pertinent to the older population, recent data indicates that ICF among older adults was associated with altered discharge destinations. In scenarios where hospitals shared health information through HIEs, beneficiaries exhibited higher odds of being discharged home with home support, showcasing the potential value of HIEs in promoting better care coordination and outcomes [43]. A recent large cohort study looking at older adults, Medicare patients with fragmented admissions, showed that when institutions shared HIEs, there was a greater chance for older patients to be discharged home with support than institutionalized in a skilled nursing facility [54]. The same research looked at a subgroup of Medicare patients with Alzheimer’s disease and found that information sharing between unrelated hospitals via a shared HIE was associated with lower in-hospital mortality [55]. In-hospital mortality during readmission to a different hospital was higher if the admission and readmission hospitals participated in different HIEs or if one or both hospitals did not participate in an HIE.

Additionally, the study’s findings regarding the increased rehospitalization costs associated with ICF underscore the importance of continuity of care. Health policy initiatives should further explore and support the implementation and expansion of HIEs, as they appear to be a viable solution to address the fragmented care transitions. This could include fostering the development of standardized care protocols and care coordination mechanisms and promoting the adoption of electronic health records to ensure seamless transitions and prevent unnecessary readmissions. In the U.S., the Health Information Technology for Economic and Clinical Health (HITECH) Act of 2009 was specifically enacted to incentivize the adoption of health information technologies and provide funding for the expansion of health information exchanges, with two of its goals being improving the quality, safety, and efficiency of care, and increasing the coordination of care. Despite having similar electronic health record technologies as in the U.S., legislation to encourage HIEs as one solution for care fragmentation and associated targeted federal funding has not yet happened in Canada [56].

The observation that delayed discharge after readmission was more likely to occur at index hospitals suggests the need for targeted interventions to address these hospitals’ specific challenges. Strategies to improve capacity, staffing, and resources at index hospitals can help mitigate the risk of delayed discharges and improve the overall efficiency of care delivery. Additionally, policy efforts should prioritize addressing the needs of older, complex patients with higher levels of frailty and comorbidity, as they face unique challenges during care transitions. Finally, the study’s emphasis on socioeconomic factors as contributors to health disparities and unequal health outcomes highlights the importance of addressing social determinants of health in health policy. Policies should focus on reducing barriers related to residency, sex, and ethnic minority status, ensuring equitable access to healthcare services, and addressing the underlying social and economic factors that contribute to health disparities. Targeted public health initiatives, outreach programs, and support services, bolstered by a robust HIE infrastructure, can help promote health equity and improve outcomes for vulnerable populations. Furthermore, given the pivotal role of ICF in healthcare outcomes, it is imperative that quality improvement programs, including Medicare’s Hospital Readmissions Reduction Program [57], integrate strategies that specifically target ICF to enhance the effectiveness of these initiatives. Overall, our findings call for a comprehensive and integrated approach to health policy that addresses interhospital care fragmentation, promotes care coordination, improves access to healthcare services, and tackles social determinants of health to achieve better patient outcomes.

### 4.4. Future works and limitations

Our study has several limitations; hence, several considerations should be considered when interpreting the results of this study. First, this was a retrospective cohort study with limited control over data collection that can be subject to selection bias. Although we have adjusted for multiple characteristics, have used propensity score matching to adjust for potential confounding variables, and performed several sensitivity analyses, it is still possible that there are other unmeasured confounding variables that we did not include in our analysis, such as treatment-related factors (e.g., type and level of care received and adherence). Thus, further studies can help validate our findings and better understand their implications in practice. Second, logistic regression can only establish associations between variables and cannot determine causality. Further studies using experimental designs, such as randomized controlled trials, may be warranted to establish causal relationships. Regarding the potential impact that HIEs may have on fragmented care in Ontario, repeating this analysis after the implementation of HIEs may highlight beneficial changes as suggested in the literature. As more Ontario hospitals introduce advanced health information systems with the need to share health information digitally, hospital networks and HIS vendors are working to establish HIEs, including those between acute care hospitals and long-term care facilities [58].

## 5. Conclusion

Our study unearthed significant factors contributing to ICF, notably discharge to facilities other than home or home care, and travel distance being the strongest risk factors. ICF escalated rehospitalization costs within 30 days, extended the LOS, and resulted in delayed discharges, especially for patients who underwent surgery during their initial hospital stay. A pronounced disparity was observed in rural residents facing a greater risk of ICF compared to urban counterparts, highlighting rural residency as a significant source of health inequality. Addressing care fragmentation and promoting equitable access to coordinated, high-quality care is important in reducing health disparities. Strategies and interventions aimed at improving care coordination, reducing barriers to care, and enhancing access to preventive services can all help to improve care coordination and reduce fragmentation, particularly for vulnerable populations. Given Ontario’s vast geographic size and population distribution, it is unlikely that patients from remote communities with complex illnesses could ever receive the totality of their care within one local hospital, thus making hospital care fragmentation unavoidable. Health information exchanges can show promise to help address gaps in health information and patient/family care goals, which may improve outcomes and increase efficiency, particularly for vulnerable older patients who cannot communicate their health stories or care goals.

## Supporting information

S1 Fig Data eligibility flow diagram. (TIF)

S1 Table Descriptive details regarding the patient’s characteristics. (DOCX)

S2 Table Risk factors associated with ICF defined based on facility. (DOCX)

S3 Table Association between ICF, defined based on facility, and delayed discharge (alternate level of care). (DOCX)

S4 Table Association between ICF, defined based on facility, and daily readmission cost. (DOCX)

S5 Table Association between ICF, defined based on facility, and prolonged length of stay. (DOCX)

S6 Table Risk factors associated with ICF: GLM vs. standardization and Doubly robust estimation. (DOCX)

S7 Table Association between ICF and patient outcomes based on matched data from propensity score matching. (DOCX)

S8 Table Odds ratios for distance across outcomes: Mean imputation vs. removal of missing data. (DOCX)

S9 Table Association between ICF and patient outcomes: Mean imputation vs. removal of missing data. (DOCX)
