# Supplementary figures and images for: Data-driven insights into interhospital care fragmentation: Implications for health policy and equity among older adults
Source: PLoS One. 2025 Feb 4;20(2):e0316829. doi: 10.1371/journal.pone.0316829 (PMC11793756; doi:10.1371/journal.pone.0316829)

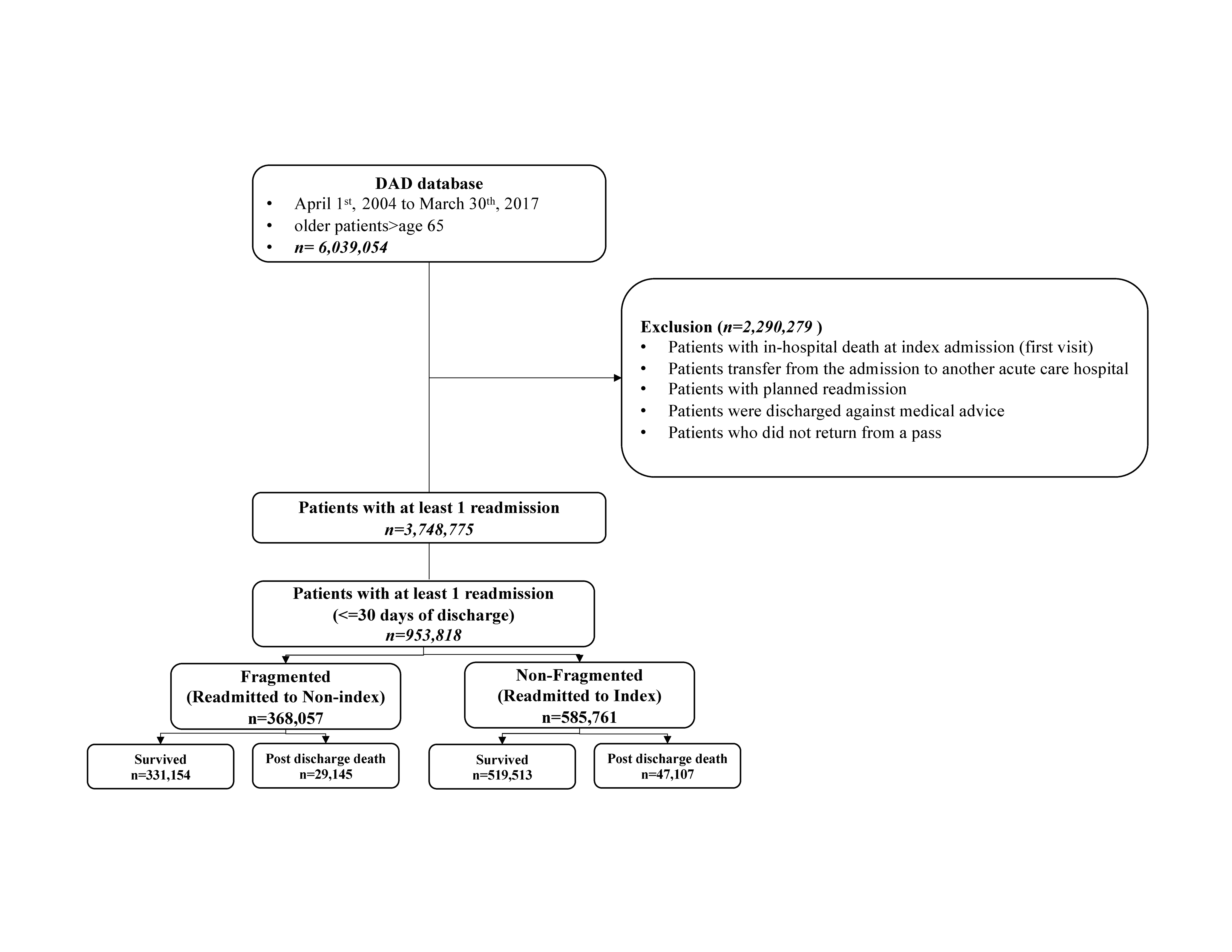

Supplement: S1 Fig — (TIF) [file pone.0316829.s001.tif]
