# Supplementary material for: Data-driven insights into interhospital care fragmentation: Implications for health policy and equity among older adults
Source: PLoS One. 2025 Feb 4;20(2):e0316829. doi: 10.1371/journal.pone.0316829 (PMC11793756; doi:10.1371/journal.pone.0316829)
Supplement: S1 Table — (DOCX) [file pone.0316829.s002.docx]

**S1 Table.** Descriptive details regarding the patient's characteristics

| **Characteristics** | **ICF (n=349,618)** | **Non-ICF(n=613,702)** | **OR**^‡^ **(95% CI)** |
| --- | --- | --- | --- |
| **Age, mean (SD)** | 74.5 (7.4) | 75.5 (7.8) | - |
| Group 1: 65-71 | 142,808 (40.8%) | 226,143 (36.8%) | Ref. |
| Group 2: 72-81 | 138,355 (39.6%) | 241,475 (39.3%) | 0.90 (0.89-0.91) |
| Group 3: 82-91 | 62,113 (17.8%) | 132,028 (21.5%) | 0.74 (0.73-0.75) |
| Group 4: >91 | 6,342 (1.8%) | 14,056 (2.3%) | 0.71 (0.69-0.73) |
| **Sex** |  |  |  |
| Male | 187,325 (53.6%) | 306,803 (50%) | Ref. |
| Female | 162,293 (46.4%) | 306,899 (50%) | 0.86 (0.85-0.87) |
| **Residency** |  |  |  |
| Urban | 257,077 (73.5%) | 507,015 (82.6%) | Ref. |
| Rural | 92,541 (26.5%) | 106,687 (17.4%) | 1.71 (1.69-1.72) |
| **Distance, mean Km (SD)** | 42 (93.3) | 18.2 (54.2) |  |
| ≤ mean | 224,131 (64.1%) | 531,959 (86.6%) | Ref. |
| > mean | 125,487 (35.9%) | 81,743 (13.3%) | 3.64 (3.60-3.68) |
| **Ethnic concentration** |  |  |  |
| Quintiles 1-4 (Majority) | 295,344 (84.5%) | 512,829 (83.6%) | Ref. |
| Quintile 5 (Minority) | 54,274 (15.5%) | 100,873 (16.4%) | 0.93 (0.92-0.94) |
| **Discharge destination** |  |  |  |
| Routine (home) | 100,219 (28.7%) | 291,656 (47.5%) | Ref. |
| Home Support | 48,361 (13.8%) | 207,231 (33.8%) | 0.68 (0.67-0.69) |
| Others | 201,038 (57.5%) | 114,815 (18.7%) | 5.09 (5.04-5.14) |
| **Comorbidity, mean (SD)** | 1.43 (1.78) | 1.52 (1.82) | - |
| 0 | 148,816 (42.6%) | 245,095 (39.9%) | Ref. |
| 1-4 | 175,954 (50.3%) | 320,270 (52.2%) | 0.90 (0.89-0.91) |
| ≥5 | 24,848 (7.1%) | 48,337 (7.9%) | 0.84 (0.83-0.86) |
| **Frailty, mean (SD)** | 2.55 (3.45) | 2.60 (3.52) | - |
| <5 | 291,313 (83.3%) | 504,381 (82.2%) | Ref. |
| 5-15 | 54,170 (15.5%) | 101,838 (16.6%) | 0.92 (0.91-0.93) |
| >15 | 4,135 (1.2%) | 7,483 (1.2%) | 0.95 (0.92-0.99) |
| **Visited SCU** |  |  |  |
| No | 245,987 (70.4%) | 526,411 (85.8%) | Ref. |
| Yes | 103,631 (29.6%) | 87,291 (14.2%) | 2.54 (2.51-2.56) |
| **Surgical Services Flag** |  |  |  |
| No | 283,575 (81.1%) | 529,195 (86.2%) | Ref. |
| Yes | 66,043 (18.9%) | 84,507 (13.8%) | 1.45 (1.44-1.47) |
| **Feeding Tube** |  |  |  |
| No | 346,720 (99.2%) | 610,158 (99.4%) | Ref. |
| Yes | 2,898 (0.8%) | 3,544 (0.6%) | 1.43 (1.36-1.51) |
| **Parenteral Nutrition** |  |  |  |
| No | 346,397 (99.1%) | 609,574 (99.3%) | Ref. |
| Yes | 3,221 (0.9%) | 4,128 (0.7%) | 1.37 (1.31-1.43) |
| **Chemotherapy** |  |  |  |
| No | 347,566 (99.4%) | 609,586 (99.3%) | Ref. |
| Yes | 2,052 (0.6%) | 4,116 (0.7%) | 0.87 (0.82-0.92) |
| **Radiotherapy** |  |  |  |
| No | 347,458 (99.4%) | 610,881 (99.5%) | Ref. |
| Yes | 2,160 (0.6%) | 2,821 (0.5%) | 1.34 (1.27-1.42) |
| **Vascular** **Access Device** |  |  |  |
| No | 332,229 (95%) | 592,478 (96.5%) _ | Ref. |
| Yes | 17,389 (5%) | 21,224 (3.5%) | 1.46 (1.43-1.49) |
| **Dialysis** |  |  |  |
| No | 342,784 (98%) | 601,621 (98%) | Ref. |
| Yes | 6,834 (2%) | 12,081 (2%) | 0.99 (0.96-1.02) |
| **Paracentesis** |  |  |  |
| No | 347,388 (99.4%) | 607,988 (99.1%) | Ref. |
| Yes | 2,230 (0.6%) | 5,714 (0.9%) | 0.68 (0.65-0.71) |
| **Pleurocentesis** |  |  |  |
| No | 334,997 (95.8%) | 604,681 (98.5%) | Ref. |
| Yes | 14,621 (4.2%) | 9,021 (1.5%) | 1.15 (1.12-1.19) |
| **Tracheostomy** |  |  |  |
| No | 347,795 (99.5%) | 612,752 (99.8%) | Ref. |
| Yes | 1,823 (0.5%) | 950 (0.2%) | 3.38 (3.12-3.65) |
| **MV (short)** |  |  |  |
| No | 343,627 (98.3%) | 604,610 (98.5%) | Ref. |
| Yes | 5,991 (1.7%) | 9,092 (1.5%) | 2.92 (2.84-3.00) |
| **MV (long)** |  |  |  |
| No | 344,363 (98.5%) | 610,727 (99.5%) | Ref. |
| Yes | 5,255 (1.5%) | 2,975 (0.5%) | 3.13 (2.99-3.27) |
| **Heart Resuscitation** |  |  |  |
| No | 348,570 (99.7%) | 613,148(99.9%) | Ref. |
| Yes | 1,048 (0.3%) | 554 (0.1%) | 3.27 (3.00-3.69) |
| **Biopsy** |  |  |  |
| No | 342,528 (98%) | 598,235 (97.5%) | Ref. |
| Yes | 7,090 (2%) | 15,467 (2.5%) | 0.80 (0.77-0.82) |
| **Endoscopy** |  |  |  |
| No | 343,134 (98.1%) | 597,531 (97.4%) | Ref. |
| Yes | 64,84 (1.9%) | 16,171 (2.6%) | 0.69 (0.67-0.71) |
| **Outcomes:** | | | |
| **Delayed Discharge** |  |  |  |
| Prevalence (%) | 11.1 | 12.4 |  |
| No | 297,178 (88.9%) | 534,640 (87.6%) | Ref. |
| Yes | 37,059 (11.1%) | 75,777 (12.4%) | 0.88 (0.87-0.89) |
| **Excessive Cost ($)** |  |  |  |
| Mean (SD) | 1992 (2021) | 1648 (1558) |  |
| No | 223,022 (63.9%) | 438,729 (71.6%) | Ref. |
| Yes | 126,243 (36.1%) | 174,447 (28.4%) | 1.42 (1.41-1.43) |
| **Prolonged LOS** |  |  |  |
| Mean (SD) | 10.8 (19.7) | 11.1 (20.3) |  |
| No | 203,020 (58.1%) | 363,387 (59.2%) | Ref. |
| Yes | 146,598 (41.9%) | 250,315 (40.8%) | 1.05 (1.04-1.06) |
| ^‡^ Unadjusted Odds Ratio  MV: mechanical ventilation; SCU: special care unit; ICF: interhospital care fragmentation | | | |
