# Supplementary material for: Data-driven insights into interhospital care fragmentation: Implications for health policy and equity among older adults
Source: PLoS One. 2025 Feb 4;20(2):e0316829. doi: 10.1371/journal.pone.0316829 (PMC11793756; doi:10.1371/journal.pone.0316829)
Supplement: S2 Table — (DOCX) [file pone.0316829.s003.docx]

## **Sensitivity Analysis 1: Changing facility number with the institution number**

**S2 Table.** Risk factors associated with ICF defined based on facility.

| **Variables** | **OR (95% CI)** |
| --- | --- |
| Age Group2 | 0.87 (0.86-0.88) |
| Age Group3 | 0.71 (0.70-0.72) |
| Age Group4 | 0.66 (0.64-0.68) |
| Sex (Female vs. Male) | 0.87 (0.86-0.88) |
| Residency (Rural vs. Urban) | 1.15 (1.14-1.17) |
| Distance (Km) | 3.11 (3.07-3.15) |
| Comorbidity score (Moderate) | 0.87 (0.87-0.88) |
| Comorbidity score (High) | 0.95 (0.93-0.97) |
| Frailty score (Moderate) | 0.67 (0.66-0.67) |
| Frailty score (High) | 0.55 (0.53-0.58) |
| Visited SCU | 1.77 (1.75-1.79) |
| Ethnic Concentration (High) | 1.27 (1.26-1.29) |
| Surgery Service | 0.89 (0.88-0.90) |
| Discharge Destination (Homecare vs. Home) | 0.78 (0.77-0.79) |
| Discharge Destination (Others vs. Home) | 4.76 (4.70-4.81) |
| Chemotherapy | 1.00 (0.94-1.07) |
| Dialysis | 0.72 (0.69-0.75) |
| Feeding Tube | 0.78 (0.73-0.83) |
| Heart Resuscitation | 1.14 (1.02-1.27) |
| Mechanical Ventilation (Long) | 0.99 (0.94-1.05) |
| Mechanical Ventilation (Short) | 1.22 (1.18-1.26) |
| Parenteral Nutrition | 0.94 (0.89-1.00) |
| Paracentesis | 0.83 (0.78-0.88) |
| Pleurocentesis | 1.10 (1.06-1.14) |
| Radiotherapy | 1.33 (1.24-1.42) |
| Tracheostomy | 1.26 (1.15-1.39) |
| Vascular Access Device | 0.91 (0.89-0.94) |
| Biopsy | 1.03 (1.00-1.07) |
| Endoscopy | 0.76 (0.74-0.79) |
