# Supplementary material for: Data-driven insights into interhospital care fragmentation: Implications for health policy and equity among older adults
Source: PLoS One. 2025 Feb 4;20(2):e0316829. doi: 10.1371/journal.pone.0316829 (PMC11793756; doi:10.1371/journal.pone.0316829)
Supplement: S3 Table — (DOCX) [file pone.0316829.s004.docx]

## **Sensitivity Analysis 1: Changing facility number with the institution number**

**S3 Table.** Association between ICF, defined based on facility, and delayed discharge (alternate level of care)

| **Variables** | **OR (95% CI)** |
| --- | --- |
| ICF | *0.75 (0.73-0.76)* |
| Age Group2 | 1.45 (1.43-1.47) |
| Age Group3 | 1.86 (1.82-1.89) |
| Age Group4 | 2.10 (2.02-2.18) |
| Sex (Female vs. Male) | 1.15 (1.13-1.16) |
| Residency (Rural vs. Urban) | 0.72 (0.71-0.73) |
| Distance (Km) | 1.21 (1.19-1.23) |
| Ethnic Concentration (High) | 0.94 (0.92-0.96) |
| Comorbidity score (Moderate) | 1.07 (1.05-1.08) |
| Comorbidity score (High) | 1.12 (1.09-1.15) |
| Frailty score (Moderate) | 1.68 (1.65-1.71) |
| Frailty score (High) | 1.85 (1.76-1.93) |
| Visited SCU | 0.75 (0.73-0.76) |
| Surgery Service | 1.18 (1.16-1.20) |
| Discharge Destination (Homecare vs. Home) | 2.06 (2.03-2.10) |
| Discharge Destination (Others vs. Home) | 1.87 (1.84-1.90) |
| Chemotherapy | 0.78 (0.72-0.85) |
| Dialysis | 1.32 (1.26-1.37) |
| Feeding Tube | 1.01 (0.94-1.09) |
| Heart Resuscitation | 0.69 (0.59-0.82) |
| Mechanical Ventilation (Long) | 1.86 (1.74-1.99) |
| Mechanical Ventilation (Short) | 1.15 (1.10-1.21) |
| Parenteral Nutrition | 0.89 (0.83-0.96) |
| Paracentesis | 0.96 (0.90-1.03) |
| Pleurocentesis | 0.95 (0.91-1.00) |
| Radiotherapy | 1.65 (1.53-1.78) |
| Tracheostomy | 1.31 (1.18-1.46) |
| Vascular Access Device | 1.07 (1.04-1.11) |
| Biopsy | 1.11 (1.06-1.16) |
| Endoscopy | 0.98 (0.94-1.03) |
| ICF: interhospital care fragmentation based on the facility | |
