# Supplementary material for: Data-driven insights into interhospital care fragmentation: Implications for health policy and equity among older adults
Source: PLoS One. 2025 Feb 4;20(2):e0316829. doi: 10.1371/journal.pone.0316829 (PMC11793756; doi:10.1371/journal.pone.0316829)
Supplement: S4 Table — (DOCX) [file pone.0316829.s005.docx]

## **Sensitivity Analysis 1: Changing facility number with the institution number**

**S4 Table.** Association between ICF, defined based on facility, and daily readmission cost.

| **Variables** | **OR (95% CI)** |
| --- | --- |
| *ICF* | *1.43 (1.42-1.45)* |
| Age Group2 | 0.87 (0.86-0.88) |
| Age Group3 | 0.75 (0.74-0.76) |
| Age Group4 | 0.69 (0.67-0.71) |
| Sex (Female vs. Male) | 0.84 (0.83-0.85) |
| Residency (Rural vs. Urban) | 0.85 (0.84-0.86) |
| Distance (Km) | 0.73 (0.72-0.74) |
| Ethnic Concentration (High) | 1.04 (1.03-1.06) |
| Comorbidity score (Moderate) | 0.94 (0.94-0.95) |
| Comorbidity score (High) | 0.79 (0.78-0.81) |
| Frailty score (Moderate) | 0.80 (0.79-0.81) |
| Frailty score (High) | 0.80 (0.77-0.83) |
| Visited SCU | 1.33 (1.31-1.34) |
| Surgery Service | 0.77 (0.76-0.78) |
| Discharge Destination (Homecare vs. Home) | 0.79 (0.78-0.80) |
| Discharge Destination (Others vs. Home) | 1.03 (1.02-1.05) |
| Chemotherapy | 1.05 (0.99-1.11) |
| Dialysis | 1.53 (1.49-1.58) |
| Feeding Tube | 1.00 (0.94-1.06) |
| Heart Resuscitation | 1.68 (1.52-1.86) |
| Mechanical Ventilation (Long) | 1.18 (1.12-1.24) |
| Mechanical Ventilation (Short) | 1.16 (1.13-1.20) |
| Parenteral Nutrition | 0.90 (0.85-0.95) |
| Paracentesis | 1.03 (0.98-1.09) |
| Pleurocentesis | 0.91 (0.88-0.94) |
| Radiotherapy | 0.67 (0.62-0.72) |
| Tracheostomy | 1.09 (0.99-1.18) |
| Vascular Access Device | 0.99 (0.97-1.02) |
| Biopsy | 0.88 (0.86-0.91) |
| Endoscopy | 0.89 (0.87-0.92) |
| ICF: interhospital care fragmentation based on the facility | |
