# Supplementary material for: Data-driven insights into interhospital care fragmentation: Implications for health policy and equity among older adults
Source: PLoS One. 2025 Feb 4;20(2):e0316829. doi: 10.1371/journal.pone.0316829 (PMC11793756; doi:10.1371/journal.pone.0316829)
Supplement: S5 Table — (DOCX) [file pone.0316829.s006.docx]

## **Sensitivity Analysis 1: Changing facility number with the institution number**

**S5 Table.** Association between ICF, defined based on facility, and prolonged length of stay.

| **Variables** | **OR (95% CI)** |
| --- | --- |
| *ICF* | *0.95 (0.94-0.97)* |
| Age Group2 | 1.19 (1.18-1.20) |
| Age Group3 | 1.24 (1.22-1.25) |
| Age Group4 | 1.21 (1.17-1.25) |
| Sex (Female vs. Male) | 1.09 (1.08-1.10) |
| Residency (Rural vs. Urban) | 0.93 (0.92-0.94) |
| Distance (Km) | 1.00 (0.98-1.01) |
| Ethnic Concentration (High) | 1.07 (1.05-1.08) |
| Comorbidity score (Moderate) | 1.16 (1.15-1.17) |
| Comorbidity score (High) | 1.24 (1.22-1.26) |
| Frailty score (Moderate) | 1.35 (1.33-1.37) |
| Frailty score (High) | 1.36 (1.31-1.41) |
| Visited SCU | 0.89 (0.88-0.90) |
| Surgery Service | 1.07 (1.05-1.08) |
| Discharge Destination (Homecare vs. Home) | 1.43 (1.42-1.45) |
| Discharge Destination (Others vs. Home) | 1.58 (1.56-1.60) |
| Chemotherapy | 1.01 (0.96-1.07) |
| Dialysis | 1.18 (1.14-1.22) |
| Feeding Tube | 1.08 (1.02-1.14) |
| Heart Resuscitation | 0.84 (0.76-0.94) |
| Mechanical Ventilation (Long) | 1.45 (1.38-1.53) |
| Mechanical Ventilation (Short) | 1.11 (1.08-1.14) |
| Parenteral Nutrition | 1.28 (1.21-1.34) |
| Paracentesis | 1.07 (1.02-1.12) |
| Pleurocentesis | 1.14 (1.10-1.18) |
| Radiotherapy | 1.33 (1.26-1.42) |
| Tracheostomy | 1.48 (1.35-1.61) |
| Vascular Access Device | 1.30 (1.27-1.33) |
| Biopsy | 1.27 (1.23-1.31) |
| Endoscopy | 1.03 (1.00-1.06) |
| ICF: interhospital care fragmentation based on the facility | |
