# Supplementary material for: Data-driven insights into interhospital care fragmentation: Implications for health policy and equity among older adults
Source: PLoS One. 2025 Feb 4;20(2):e0316829. doi: 10.1371/journal.pone.0316829 (PMC11793756; doi:10.1371/journal.pone.0316829)
Supplement: S6 Table — (DOCX) [file pone.0316829.s007.docx]

## **Sensitivity Analyses 2 and 3: Reducing the Effects of Confounding**

**S6 Table.** Risk factors associated with ICF: GLM vs. standardization and Doubly Robust estimation

| **Variables** | **GLM** | **Reg. Standardization** | **DRE** |
| --- | --- | --- | --- |
| Age Group2 | 0.90 (0.89-0.91) | 0.92 (0.91-0.93) | 0.91 (0.90-0.92) |
| Age Group3 | 0.74 (0.73-0.75) | 0.78 (0.77-0.79) | 0.79 (0.78-0.80) |
| Age Group4 | 0.70 (0.68-0.73) | 0.75 (0.73-0.77) | 0.71 (0.68-0.73) |
| Sex (Female vs. Male) | 0.87 (0.87-0.88) | 0.90 (0.89-0.91) | 0.90 (0.89-0.91) |
| Residency (Rural vs. Urban) | 1.11 (1.10-1.12) | 1.12 (1.11-1.13) | 1.17 (1.16-1.18) |
| Distance (Km) | 3.50 (3.45-3.54) | 2.45 (2.42-2.48) | 2.30 (2.27-2.33) |
| Comorbidity score (Moderate) | 0.88 (0.87-0.89) | 0.91 (0.90-0.92) | 0.90 (0.89-0.91) |
| Comorbidity score (High) | 0.96 (0.94-0.98) | 0.96 (0.94-0.98) | 0.94 (0.92-0.96) |
| Frailty score (Moderate) | 0.71 (0.70-0.72) | 0.76 (0.75-0.77) | 0.70 (0.69-0.71) |
| Frailty score (High) | 0.62 (0.59-0.65) | 0.68 (0.65-0.71) | 0.61 (0.58-0.63) |
| Visited SCU | 1.86 (1.84-1.89) | 1.69 (1.67-1.70) | 1.52 (1.48-1.55) |
| Ethnic Concentration (High) | 1.27 (1.25-1.29) | 1.20 (1.18-1.21) | 1.26 (1.24-1.27) |
| Surgery Service | 0.99 (0.98-1.01) | 1.02 (1.01-1.03) | 0.99 (0.98-1.00) |
| Discharge Destination (Homecare vs. Home) | 0.78 (0.77-0.79) | 0.78 (0.77-0.79) | 0.71 (0.70-0.72) |
| Discharge Destination (Others vs. Home) | 5.71 (5.65-5.77) | 4.90 (4.85-4.95) | 4.77 (4.71-4.84) |
| Chemotherapy | 1.00 (0.94-1.06) | 1.02 (0.98-1.07) | 1.12 (0.94-1.34) |
| Dialysis | 0.76 (0.74-0.79) | 0.82 (0.80-0.84) | 0.88 (0.82-0.96) |
| Feeding Tube | 0.74 (0.7-0.79) | 0.79 (0.75-0.83) | 0.84 (0.76-0.93) |
| Heart Resuscitation | 1.22 (1.09-1.37) | 1.17 (1.06-1.27) | 1.21 (1.08-1.35) |
| Mechanical Ventilation (Long) | 1.10 (1.04-1.17) | 1.09 (1.04-1.14) | 1.29 (1.25-1.33) |
| Mechanical Ventilation (Short) | 1.29 (1.25-1.33) | 1.23 (1.20-1.26) | 1.13 (1.07-1.19) |
| Parenteral Nutrition | 0.92 (0.87-0.98) | 0.93 (0.89-0.97) | 0.92 (0.87-0.96) |
| Paracentesis | 0.80 (0.76-0.85) | 0.83 (0.79-0.87) | 0.76 (0.71-0.82) |
| Pleurocentesis | 1.07 (1.03-1.12) | 1.05 (1.02-1.09) | 1.04 (1.00-1.07) |
| Radiotherapy | 1.35 (1.27-1.45) | 1.29 (1.23-1.36) | 1.34 (1.27-1.42) |
| Tracheostomy | 1.32 (1.19-1.45) | 1.25 (11.15-1.35) | 1.33 (1.21-1.46) |
| Vascular Access Device | 0.92 (0.90-0.95) | 0.94 (0.92-0.96) | 0.91 (0.89-0.94) |
| Biopsy | 1.08 (1.04-1.11) | 1.06 (1.03-1.08) | 1.07 (1.04-1.11) |
| Endoscopy | 0.77 (0.75-0.8) | 0.81 (0.78-0.83) | 0.69 (0.67-0.72) |
| *Reg. standardization: Regression Standardization; DRE**: Doubly Robust Estimation* | | | |
