# Supplementary material for: Data-driven insights into interhospital care fragmentation: Implications for health policy and equity among older adults
Source: PLoS One. 2025 Feb 4;20(2):e0316829. doi: 10.1371/journal.pone.0316829 (PMC11793756; doi:10.1371/journal.pone.0316829)
Supplement: S7 Table — (DOCX) [file pone.0316829.s008.docx]

## **Sensitivity Analyses 2 and 3: Reducing the Effects of Confounding**

**S7 Table.** Association between ICF and patient outcomes based on *Matched Data* from propensity score matching.

| **Outcome** | **OR (95% CI)** | |
| --- | --- | --- |
|  | **Original Data** | **Matched Data (1:1)** |
| Delayed Discharge | 0.87 (0.86-0.88) | 0.91 (0.88-0.94) |
| Daily Costs | 1.36 (1.34-1.37) | 1.30 (1.27-1.33) |
| Prolonged length of stay | 0.99 (0.98-1.00) | - 1. (1.00-1.06) |
