# Supplementary material for: Data-driven insights into interhospital care fragmentation: Implications for health policy and equity among older adults
Source: PLoS One. 2025 Feb 4;20(2):e0316829. doi: 10.1371/journal.pone.0316829 (PMC11793756; doi:10.1371/journal.pone.0316829)
Supplement: S8 Table — (DOCX) [file pone.0316829.s009.docx]

## **Sensitivity Analysis 4: Addressing missing values**

**S8 Table.** Odds Ratios for distance across outcomes: mean imputation vs. removal of missing data

| Outcome | **OR (95% CI)** for Distance | |
| --- | --- | --- |
|  | **Imputed by mean** | **Removed missing** |
| ICF | 3.49 (3.45-3.53) | 3.22 (3.18-3.26) |
| Delayed Discharge | 1.175 (1.154-1.96) | 1.176 (1.155-1.197) |
| Daily Costs | 0.740 (0.731-0.750) | 0.736 (0.727-0.746) |
| Prolonged length of stay | 0.976 (0.965-0.987) | 0.962 (0.951-0.973) |
