# Supplementary material for: Data-driven insights into interhospital care fragmentation: Implications for health policy and equity among older adults
Source: PLoS One. 2025 Feb 4;20(2):e0316829. doi: 10.1371/journal.pone.0316829 (PMC11793756; doi:10.1371/journal.pone.0316829)
Supplement: S9 Table — (DOCX) [file pone.0316829.s010.docx]

## **Sensitivity Analysis 4: Addressing missing values**

**S9 Table.** Association between ICF and Patient Outcomes: mean Imputation vs. removal of missing data

| **Outcome** | **OR (95% CI)** | |
| --- | --- | --- |
|  | **Imputed by mean** | **Removed missing** |
| Delayed Discharge | 0.87 (0.86-0.88) | 0.87 (0.86-0.88) |
| Daily Costs | 1.357 (1.343-1.371) | 1.352 (1.338-1.366) |
| Prolonged length of stay | 0.984 (0.975-0.994) | 1.001 (1.000-1.002) |

We also replaced the missing data with the median as an alternative approach for handling missing values. The results remained unchanged after converting the continuous variable to a factor variable. The data distribution for this variable did not shift significantly due to the small proportion of missing values. The mean changed slightly from 26.86 to 26.42, the 3^rd^ quartile shifted from 21.9 to 19.63, and the median moved from 7.28 to 7.005, with no minimum and maximum values change. Since the categorical version of the variable was included in our model and the categorical distribution remained stable, the results were robust even after median imputation.
